# Hot Drinks and Oesophageal Cancer: Prospective Analyses in Around 1 Million UK Adults

**DOI:** 10.1002/ijc.70654

**Published:** 2026-09-08

**Authors:** Keren Papier, Kezia Gaitskell, Sarah Floud, Gillian K. Reeves

**Affiliations:** ^1^ Cancer Epidemiology Unit, Nuffield Department of Population Health University of Oxford Oxford UK

**Keywords:** cohort study, hot beverages, hot drinks, oesophageal cancer, risk

## Abstract

Studies in Asia, Africa, South America and the Middle East have consistently shown that tea or mate drunk at very high temperatures (~70°C) increases oesophageal cancer risk, but evidence is limited for drink temperatures typical of Western populations. In analyses of two large UK cohorts (977,282 participants), we used Cox regression to estimate hazard ratios (hereafter referred to as RRs) and 95% confidence intervals (CIs) for oesophageal cancer subtypes by self‐reported preferred drink temperature, our primary focus and frequency of hot drink (tea and coffee) consumption. Pooled estimates were calculated using inverse‐variance‐weighted averages of cohort‐specific log RRs. During an average follow‐up of 14.2 years (Million Women Study [MWS]) and 11.1 years (United Kingdom Biobank [UKB]), 2348 incident oesophageal cancers occurred (1045 squamous cell carcinoma [SCC], 1303 adenocarcinoma [AC]). Consumption of drinks at a ‘very hot’ versus ‘warm’ temperature was associated with a 3‐fold greater SCC risk (RR = 3.17 [95% CI: 2.49, 4.03]) but not with AC risk (0.92 [0.76, 1.12]), after adjustment for consumption frequency. After controlling for drink temperature, daily consumption of 6+ versus < 6 hot drinks was associated with a 71% increase in SCC risk (1.71 [1.51, 1.94]), with little association with AC risk (1.19 [1.06, 1.33]). In UK adults, an estimated 14% of SCC oesophageal cancers could be avoided if individuals who reported consuming their drinks at a ‘very hot’ temperature instead consumed them at a temperature more representative of the temperature range described here as ‘hot’. The fact that we observed a clear trend in risk across self‐reported temperature categories suggests that further limiting the temperature at which individuals consume their drinks to something well below average for this population may well avoid an even greater proportion of SCC cases. This large UK study of middle‐aged adults, amongst whom hot drinks are widely consumed, provides clear evidence that temperature and frequency of hot drink consumption represent useful targets for oesophageal SCC prevention.

## What's New?

Hot drink consumption is widespread, but its contribution to oesophageal cancer risk at temperatures typical of Western populations remains unclear. The authors of the present study analysed data from nearly 980,000 adults in the United Kingdom to examine self‐reported drink temperature and consumption frequency in relation to oesophageal cancer risk. Drinking beverages at very hot temperatures and consuming six or more hot drinks daily were both strongly associated with an increased risk of oesophageal squamous cell carcinoma (SCC), but neither were materially associated with adenocarcinoma risk. The findings suggest that reducing drink temperature could help prevent a substantial proportion of SCC cases.

## Graphical abstract

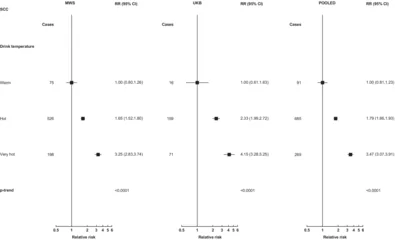

**Abbreviations**
AC: adenocarcinoma
BMI: Body mass index
CI: Confidence interval
CKB: China Kadoorie Biobank
IARC: International Agency for Research on Cancer
ICD: International Statistical Classification of Diseases
ICDO: International Statistical Classification of Diseases for Oncology
MHT: Menopausal hormone therapy
MWS: Million Women Study
NHS: National Health Service
RR: relative risk
SCC: squamous cell carcinoma
UKB: United Kingdom Biobank study

## 1 Introduction

Early case–control studies, largely conducted in Iran and South America, have consistently shown that consumption of drinks such as tea and mate at very hot temperatures (typically ~70°C) is associated with an increased risk of oesophageal cancer [1, 2]. Following a review of the available evidence in 2016, the International Agency for Research on Cancer classified ‘very hot beverages’ (defined as > 65°C) as ‘probably carcinogenic to humans’ (Group 2A) based on limited evidence for oesophageal cancer; however, there was no conclusive evidence for drinking beverages at lower temperatures [3]. Importantly, this threshold reflects a pragmatic categorisation used for evaluation purposes rather than a clear biological cut‐off for carcinogenicity. This supports the view that higher beverage temperatures, rather than their constituents, are likely to be responsible for the increased risk of oesophageal cancer.

There is considerably less evidence regarding the relevance of drink temperature for oesophageal cancer risk within the range at which hot beverages, such as tea and coffee, are usually drunk in Western populations, estimated to be around 60°C [4, 5]. In addition, few prospective studies have been sufficiently large to reliably assess the relationship between consumption of hot drinks and risk of the two main histological subtypes of oesophageal cancer, squamous cell carcinoma (SCC) and adenocarcinoma (AC) [6, 7, 8, 9, 10, 11, 12], which are both relatively common in Western populations. This is particularly important given that these two subtypes have very distinct aetiologies [13, 14], and that different cell types might plausibly be more or less affected by thermal damage. There is also limited information as to whether hot drink consumption may exacerbate the effects of other known carcinogens for oesophageal cancer such as tobacco [12]. Given the almost ubiquitous consumption of tea and coffee in Western populations [15], any impact of moderately hot drink intake on oesophageal cancer risk in such populations may be considerable.

In this study, we examine the associations between preferred drink temperature and frequency of hot drink consumption and the risks of SCC and AC of the oesophagus, using combined data on almost 980,000 participants from two large UK cohort studies, the Million Women Study (MWS) and UK Biobank.

## 2 Methods

### 2.1 Datasets

This study used data from two large UK cohorts: the Milion Women Study (MWS) and UK Biobank (UKB). Between 1996 and 2001, the MWS recruited 1.3 million women in England and Scotland through the National Health Service (NHS) Breast Screening Programme. Women aged 50–64 were sent a questionnaire, with their invitation to routine screening, which asked about sociodemographic, anthropometric, lifestyle and health factors. They were resurveyed at approximately 3–5‐year intervals thereafter. Women were followed up for deaths, cancer registrations and hospital admissions, via electronic linkage to routinely collected NHS data. In UKB, 503,317 men and women aged 37–73 years who were registered with the NHS in England, Wales and Scotland, were recruited between 2006 and 2010 by attending an in‐person baseline assessment. Information on lifestyle and sociodemographic factors, and health, was collected at recruitment via a touchscreen questionnaire, and participants were followed up for health‐related outcomes via electronic linkage to routinely collected NHS data. Further details of the MWS and UKB study protocols, methods, and questionnaires, have been published [16, 17, 18] and the questionnaires can be viewed on the study websites [19, 20]. Further details of the analysis sample are shown in the Supplementary Materials and Methods and in Figure S1.

### 2.2 Assessment of Hot Drinks

Participants in the MWS (at the 8‐year re‐survey) and UKB (at baseline) were asked similar questions regarding hot drink consumption. They were asked how they preferred/liked to drink their hot drinks with possible responses including: ‘very hot’, ‘hot’, ‘warm’, ‘do not drink hot drinks’ and ‘prefer not to answer’. Participants in both cohorts were also asked how many cups of tea and coffee they consumed each day which were summed to give their total hot drinks intake and categorised as ‘fewer than 6 hot drinks per day’, and ‘6 or more hot drinks per day’.

### 2.3 Assessment of SCC and AC

All study participants were followed through electronic linkage to centrally held National Health Service (NHS) records, which provide information on date of incident cancers and death, with cancer site and histology coded to the International Classification of Disease, 10th revision (ICD‐10) and International Classification of Diseases for Oncology, 3rd Edition ICD‐O. Our main outcomes of interest included: oesophageal cancer (ICD‐10 C15) which we divided into SCC (ICD‐O codes 8050–8086) and AC (ICD‐O codes 8140–8145, 8190–8231, 8260–8263, 8310, 8315, 8480–8490, 8570–8575).

### 2.4 Statistical Analysis

In each cohort, participants contributed person‐years from baseline (age at recruitment) until age at cancer diagnosis (except C44), emigration, date of death or end of follow‐up (censored at 31 December 2022 for MWS and 31 December 2020 [England], 30 November [Scotland] and 31 December 2016 [Wales] for UKB), whichever came first. For each study, Cox proportional hazards regression models estimated adjusted hazard ratios (RRs) and 95% confidence intervals (CIs) for oesophageal SCC and AC according to preferred hot drink temperature (warm, hot, very hot). Study‐specific RRs were pooled using inverse‐variance weighting, with heterogeneity (p‐het) assessed using chi‐square tests. Tests for linear trend in the log RRs were performed with categories coded in an ordinal fashion using a Wald test. We also estimated RRs and 95% CIs according to number of hot drinks consumed daily (< 6, 6+) within categories of preferred drink temperature for each cohort, and for both studies combined. We assessed whether the association of drink frequency with risk varied by drink temperature by conducting a chi‐square test for heterogeneity in the RRs associated with consumption of < 6 versus 6+ drinks across the three temperature categories. To estimate the independent associations of drink temperature and frequency, we estimated relative risks for oesophageal SCC and AC by temperature and daily intake, with mutual adjustment for each factor. Where results from analyses involving comparison of risks across more than two categories are presented graphically, variances are estimated by treating the log‐RRs as group‐specific log‐risks [21]. Results in the text that refer to a specific comparison between two categories are presented in the form of conventional RRs and their corresponding confidence intervals.

### 2.5 Sensitivity Analyses

To assess the potential impact on our findings of residual confounding, we calculated the change in the log RR after differing levels of adjustment for potential confounders. To assess potential reverse causality, we examined the impact of excluding the first 5 years of follow‐up. We also investigated whether adding milk modified associations between preferred tea and coffee temperature and oesophageal cancer risk in participants who completed the MWS 12‐year resurvey in which added milk intake was assessed, using likelihood ratio tests to test for interaction. To assess consistency across key subgroups, we examined pooled estimates of the mutually adjusted associations of preferred drink temperature (very hot vs. warm) and hot drink intake (6+ vs. < 6 per day) with oesophageal cancer risk across 12 personal and lifestyle characteristics, by subtype.

To quantify the possible impact of reducing the temperature at which hot drinks are consumed in a UK population, we estimated the proportion of oesophageal cancers of a given subtype that would be prevented by reducing drink temperature to a maximum of ‘hot’, a preference that was common in both populations. Estimates were based on observed patterns of preferred drink temperature in UKB which includes men and women, and pooled estimates of the association of drink temperature with risk presented here. We also translated this population attributable fraction into estimated absolute numbers of cancers that would be prevented annually, based on national incidence rates of oesophageal SCC cancer in the UK population (See Supplementary Materials and Methods for detail).

## 3 Results

In total, our analysis included 510,112 participants from MWS and 467,170 from UKB. Table 1 presents characteristics of participants in each cohort according to the number of hot drinks they consumed daily, and their preferred drink temperature. In both cohorts, just under half of participants (43% in MWS, 44% in UKB) reported consuming 6+ hot drinks per day and ~15% of participants (13% in MWS, 17% in UKB) reported a preference for consuming drinks at a very hot temperature.

**TABLE 1:** Baseline characteristics of 510,112 MWS participants and 467,170 UKB participants.

	Number of hot drinks		Drink temperature	
	0–5	6+	Hot or warm	Very hot
MWS characteristics	*N* = 289,972	*N* = 220,140	*N* = 442,831	*N* = 67,281
Age at study baseline	64.4 (4.9)	64.5 (4.8)	64.5 (4.9)	64.1 (4.8)
Most deprived	14.5%	15.9%	15.1%	15.3%
Educational attainment, tertiary	18.0%	15.2%	16.7%	17.4%
Strenuous exercise frequency, rarely/never	39.9%	42.9%	41.1%	41.7%
Smoking status, current	9.1%	16.4%	12.4%	11.1%
Alcohol drinks per week, 7+	31.6%	27.4%	29.4%	32.6%
Height in cm, 165+	38.9%	39.0%	38.6%	41.3%
BMI, kg/m^2^, 30+	16.8%	17.5%	17.5%	14.8%
MHT use, ever	53.6%	51.0%	52.4%	53.2%
Parity, nulliparous	11.3%	10.7%	11.2%	10.1%
Percentage of hot drinks coming from tea, 75%+	44.5%	47.7%	45.7%	47.0%
Number of hot drinks				
< 6	—	—	57.8%	50.4%
6+	—	—	42.2%	49.6%
Drink temperature				
Warm or hot	88.3%	84.8%	—	—
Very hot	11.7%	15.2%	—	—
UKB characteristics	*N* = 262,730	*N* = 204,440	*N* = 386,741	*N* = 80,429
Age at study baseline	56.1 (8.3)	56.7 (7.9)	56.5 (8.1)	55.6 (8.0)
Most deprived	20.6%	18.8%	19.6%	20.6%
Educational attainment, tertiary	59.9%	58.8%	59.9%	57.3%
Physical activity level, low	19.2%	18.9%	19.1%	18.9%
Smoking status, current	8.2%	13.8%	10.6%	11.0%
Alcohol, 10+ g/day	48.1%	49.1%	48.6%	48.5%
Height in cm, highest sex‐specific quintile	17.7%	18.2%	17.6%	19.3%
BMI, kg/m^2^, 30+	24.1%	25.1%	24.9%	22.4%
MHT use in women, ever	36.6%	39.6%	38.2%	36.1%
Parity in women, nulliparous	19.9%	16.8%	18.8%	17.6%
Percentage of hot drinks coming from tea, 75%+	43.3%	44.5%	43.0%	47.7%
Number of hot drinks				
< 6	—	—	57.6%	49.8%
6+	—	—	42.4%	50.2%
Drink temperature				
Warm or hot	84.7%	80.3%	—	—
Very hot	15.3%	19.7%	—	—

Abbreviation: MHT, menopausal hormone therapy.

During an average follow‐up of 14.2 (SD 4.1) years in the MWS, there were 799 cases of oesophageal SCC and 617 cases of oesophageal AC, and over an average follow‐up of 11.1 (SD 2.5) years in UKB, the corresponding numbers of cases were 246 and 686, respectively. In both cohorts, the relative risk of SCC of the oesophagus increased with increasing temperature of drink consumption, with pooled RRs in those who preferred to consume hot and very hot drinks of 1.79 (1.44, 2.23) and 3.47 (2.73, 4.40), respectively, compared with those who preferred to consume warm drinks (Figure 1). Drink temperature was associated with a higher risk of SCC in a log linear fashion (p‐trend < 0.0001 for MWS, UKB and pooled). In contrast, there was no evidence of any association between preferred drink temperature and risk of oesophageal AC in either cohort, or based on the pooled results from the two cohorts. The magnitudes of these associations were similar in both cohorts (Chi square (2df) and p‐het value comparing cohorts: SCC: *χ* ^2^ 2df 2.06, p‐het = 0.37, AC: *χ* ^2^ 2df 0.46, p‐het = 0.79).

**FIGURE 1:**
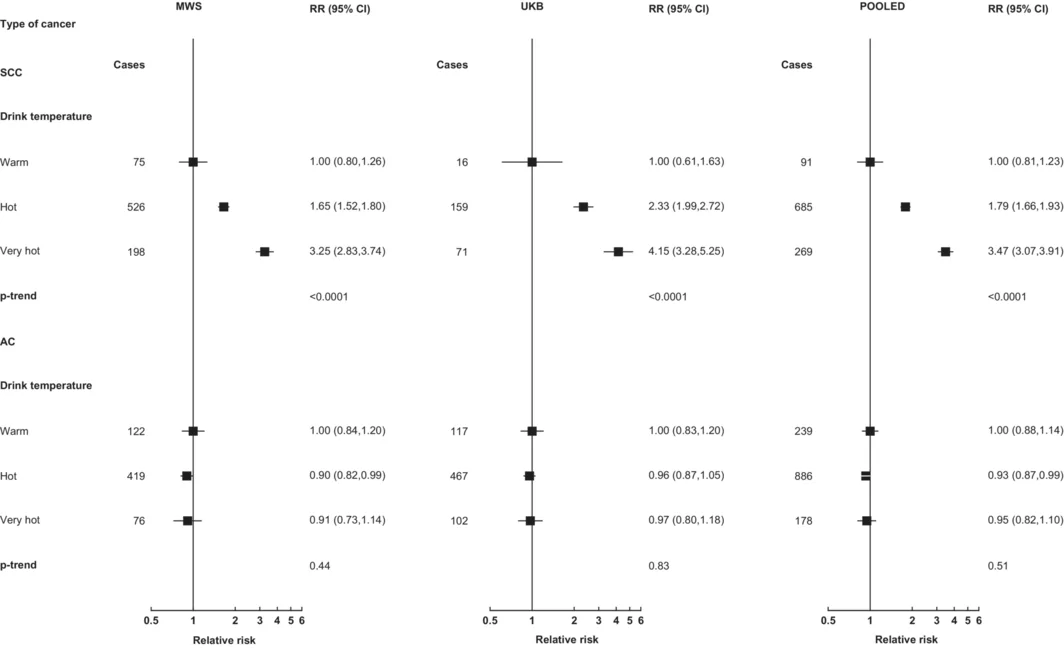
Relative risks of oesophageal squamous cell carcinoma (SCC), and oesophageal adenocarcinoma (AC) by preferred drink temperature in MWS (*N* = 510,112) and UKB (*N* = 467,170) participants.

When oesophageal cancer risk was assessed in relation to number of hot drinks consumed per day within categories of preferred drink temperature, there was evidence of an increase in SCC risk with increasing number of hot drinks per day (Figure 2) but no evidence of an interaction between drink temperature and number of hot drinks with SCC (*p* = 0.57) or AC (*p* = 0.09).

**FIGURE 2:**
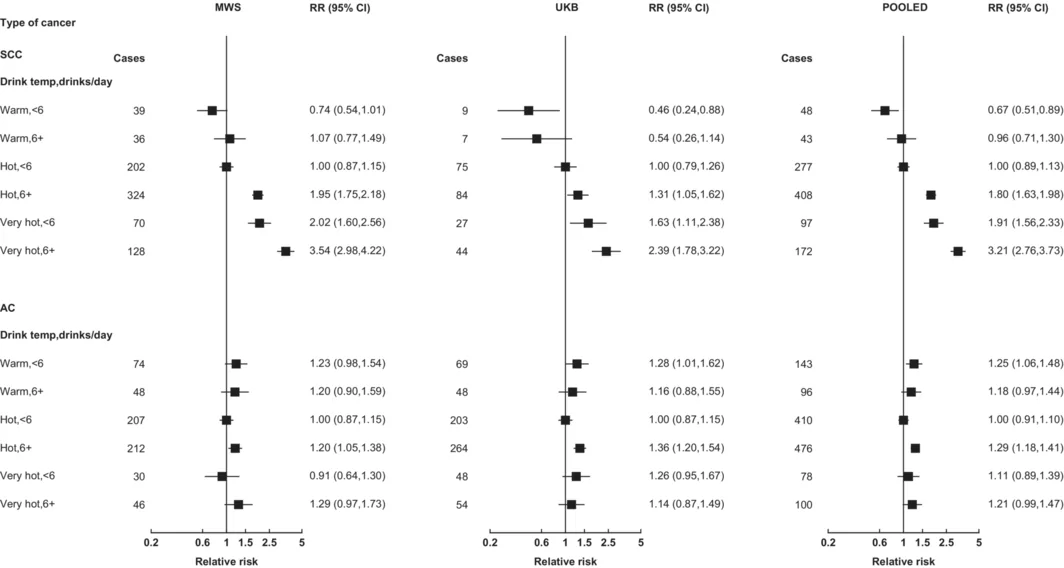
Relative risks of oesophageal squamous cell carcinoma (SCC), and oesophageal adenocarcinoma (AC) by preferred drink temperature and number of drinks consumed in MWS (*N* = 510,112) and UKB (*N* = 467,170) participants.

Incremental adjustment for relevant potential confounders did not materially change the log RRs for associations of drink temperature or number of hot drinks with SCC (Figure S2). Findings were broadly similar in analyses restricted to less than 5 years and 5 or more years after baseline for both drink temperature (very hot vs. warm pooled RR, 95% CI follow up < 5 years: 3.85 [2.25, 6.58] and 5+ years: 3.16 [2.40, 4.14]) and number of hot drinks (6+ vs. < 6 pooled RR < 5 years 1.40 [1.07, 1.84] and 5+ years 1.78 [1.54, 2.06]).

In 459,739 MWS participants who provided information on whether they added milk to their tea or coffee at the 12‐year resurvey, the vast majority reported adding milk to their tea (73%) and coffee (64%), with little evidence of any difference in the magnitude of the temperature‐associated risks according to whether women did or did not add milk to their tea or coffee (tea: p‐het = 0.20, coffee: p‐het = 0.32) (Figure S3).

Pooled estimates of the independent associations of preferred drink temperature and number of drinks consumed per day with the risk of oesophageal SCC, overall and according to 12 selected characteristics, are shown in Figure 3. The pooled RRs (for SCC) associated with drinking very hot and hot drinks, compared with warm drinks, remained substantially elevated after adjusting for number of hot drinks consumed (very hot RR = 3.17 [2.49, 4.03]; hot RR = 1.69 [1.36, 2.11]) with no evidence of any difference in this RR across subgroups defined by these characteristics (p‐het > 0.10 for all 12). The corresponding pooled RR for very hot for AC was 0.92 (0.76, 1.12). The pooled RR (for SCC) associated with drinking 6+ versus < 6 drinks per day, after controlling for drink temperature, was 1.71 (1.51, 1.94). This was also consistent across all subgroups considered here (p‐het > 0.05 in all cases) except for sex (p‐het = 0.004); however, this may well be due to chance given that the results for men are necessarily based only on UKB data and therefore on substantially fewer cases. The corresponding pooled RR for AC was 1.19 (1.06, 1.33).

**FIGURE 3:**
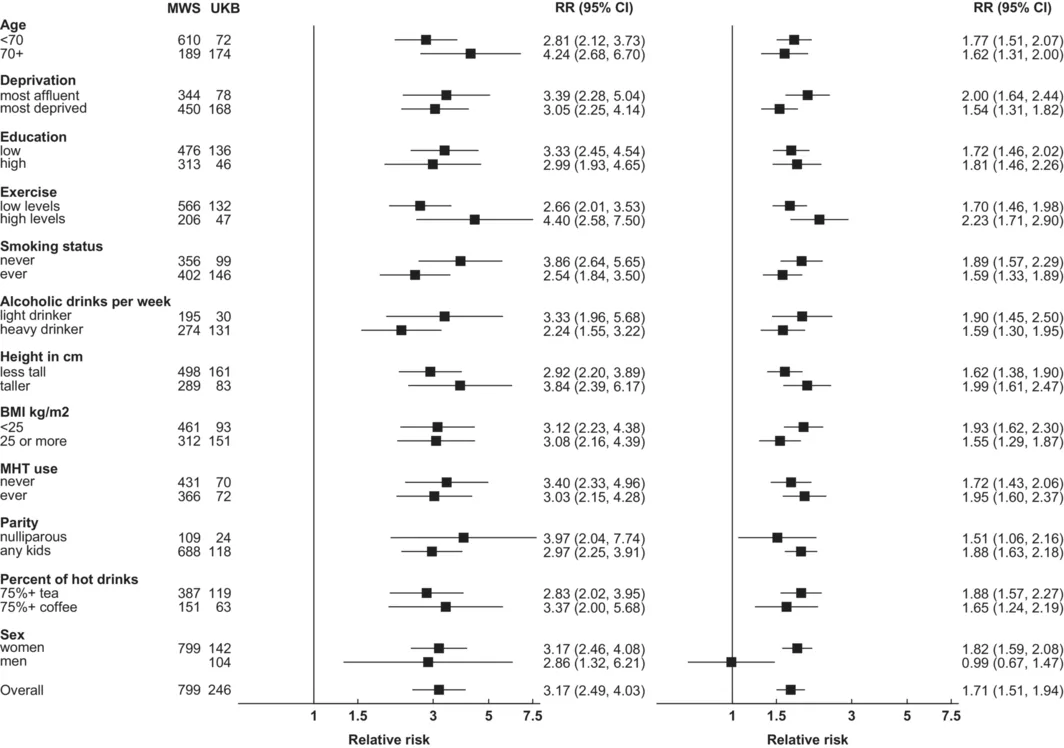
Risk of oesophageal squamous cell carcinoma by preferred drink temperature* (very hot vs. warm) and number** of drinks consumed (6+ vs. < 6) in subgroups defined by selected characteristics among MWS and UKB participants (pooled). *Adjusted for number of hot drinks. **Adjusted for drink temperature. In MWS: Deprivation (low [lowest 2 fifths of Townsend deprivation score], high [top 3 fifths]), education (low [none], high [any]), exercise (low [strenuous exercise once or less per week], high [strenuous exercise more than once per week]), height (less tall [< 163 cm], taller [163 cm or above]), alcohol (light drinker [1–6 units per week], heavy [7 or more units per week]). In UKB: Deprivation (low [lowest 2 fifths of Townsend deprivation score], high [top 3 fifths]), education (low [secondary], high [tertiary or university]), exercise (low [low or medium], high [high]), height (less tall [bottom 3 fifths of sex‐specific quintiles], taller [top 2 fifths]), alcohol (light drinker [1–9 g/day], heavy [10 or more g/day]).

Assuming a causal association between preferred drink temperature and the risk of oesophageal SCC, the estimated proportion of oesophageal SCC cancers that would be prevented if individuals who consumed drinks at a very hot temperature instead consumed their drinks at a hot temperature is 14%. Based on national incidence rates for SCC oesophageal cancers in the United Kingdom, these attributable risks translate into absolute numbers of cancers that could be prevented each year of 440.

## 4 Discussion

In this large study including over 1000 SCC cases, 1300 AC cases, and nearly 1 million adults, higher preferred drink temperature and increased frequency of hot drink consumption were each associated with a higher risk of SCC but neither was materially associated with the risk of AC. The association of drink temperature and frequency with SCC risk was similar regardless of whether individuals primarily drank tea or coffee.

Four previous prospective studies have examined drink temperature and oesophageal cancer risk by histological subtype, only one in a Western cohort. The latter study, conducted in UKB, was based on a similar number of cases as that contributed by UKB to our pooled analysis (representing ~1/3 of the total SCC cases), and its results were consistent with those reported here [11]. Another study, in the Golestan cohort in Iran, found that individuals who reported a preference for very hot tea had a two‐fold (RR = 2.41; 1.27, 4.56) higher risk of SCC compared with those who preferred cold or lukewarm tea (317 cases of SCC), which is also consistent with our findings. However, the latter study did not find an association by amount consumed and did not assess associations with AC (11 cases) [10]. An analysis in men and women in the China Kadoorie Biobank (CKB) cohort [9] with 1731 oesophageal cancer cases (the vast majority of which would have been SCC) found that compared to men who consumed tea less than once a week, those who consumed tea daily and preferred their tea temperature warm, hot or burning hot had 17%, 30% and 55% higher risks of oesophageal cancer, respectively. The study did not find this association in women, though few women consumed tea once a week or more in the CKB study. Unlike the present study, the number of cups consumed per day was not associated with oesophageal cancer risk after adjusting for drink temperature in men or women. Lastly, a large Japanese cohort study based on a census population [12], which included 440 oesophageal cancer deaths (of which SCC is likely to have accounted for 70% of cases) found that drinking green tea at hot temperatures was associated with a 60% higher risk of mortality of oesophageal cancer compared with drinking it at moderate temperatures. Although all of these studies identified an increase in the risk of SCC with greater drink temperature, not all of them were able to identify an independent effect of drink frequency, and apart from the earlier study in UKB, most were conducted in populations where the types of hot drinks consumed, and the temperatures at which they were consumed, are specific to the respective regions, limiting generalisability to Western populations. Our study has convincingly demonstrated that differences in the temperature and frequency of tea and coffee consumption, within the ranges commonly observed in the UK population, are materially associated with SCC risk.

In keeping with our findings, and those from prospective cohort studies in China, Iran and Japan cited above, case–control studies conducted within the African SCC corridor (including Kenya, Tanzania and Malawi) likewise reported an elevated risk of SCC associated with higher self‐reported beverage temperatures [22, 23]. Notably, a higher risk was observed not only with higher reported drinking temperatures but also in conjunction with more frequent mouth burning, faster drinking speed and shorter waiting times prior to consumption [22]. These observations highlight the variability in real‐world thermal exposures and underscore the limitations of relying on fixed temperature thresholds, such as 65°C, to characterise risk. Collectively, evidence across diverse populations with varying hot beverage practices strengthens the inference that thermal exposure plays a contributory role in the aetiology of SCC.

The associations of preferred drink temperature and frequency of hot drink intake with risk observed here were relatively unaffected by adjustment for key confounders, including smoking status or alcohol intake, and were evident in both never smokers and light drinkers and are therefore unlikely to be explained by residual confounding. This is in line with evidence from a large study in Japan which found an increased risk of SCC mortality associated with drinking green tea at a very hot versus moderate temperature (RR = 1.7, 1.1–2.6) among never smokers who drank alcohol infrequently (< 3 times/week or less) [12]. Although no association was observed among never smokers in the CKB, fewer than 10% of cases in that group consumed hot drinks daily, and so power was limited [9]. Furthermore, the fact that the main findings did not vary materially by follow‐up period, in line with the CKB study [9], suggests that the associations are not due to reverse causation.

The source of hot drinks did not modify the association with oesophageal SCC, suggesting that beverage temperature rather than drink constituents is the key risk factor. Animal studies support this, showing that very hot water (70°C) promotes premalignant lesions and oesophageal necrosis, whereas lower‐temperature beverages do not [24, 25]. One factor which might be expected to be associated with drink temperature is the addition of milk to tea and coffee and, if consumption of milk itself was related to risk, this could act as a potential confounder in the relationship between drink temperature and oesophageal cancer risk. However, the fact that the associations of drink temperature with risk observed here were similar in those who did and did not add milk to their hot drinks suggests that the findings are unlikely to be explained by differences in milk intake. The most likely mechanism is thermal injury, which may impair mucosal integrity and promote carcinogenic processes [24, 26, 27]. We clearly demonstrated that any association with drink temperature was confined to SCC. This observation would appear to support a thermal injury mechanism since such cancers typically arise from squamous cells in the upper two‐thirds of the oesophagus [28] which would be expected to be most susceptible to the effects of hot liquids.

With 1000 confirmed cases of SCC and ~1300 cases of AC among nearly 1 million adults across both cohorts, ours is the largest study to date, and substantially advances current understanding of the potential carcinogenic impact of patterns of hot drink consumption that are typical of Western populations. A limitation of our study was that although we examined associations by self‐reported preferred drink temperature, we did not have data on directly measured drink temperatures and therefore cannot determine how self‐reported drink temperature is likely to relate to absolute drink temperature. It is also likely that subjective perceptions of temperature vary to some extent between populations, between individuals within populations, and over time, and may therefore not be consistently interpreted. Findings from the few published studies that have examined absolute drink temperature in comparable populations to that included here suggest that tea and coffee are commonly consumed at temperatures of approximately 60°C in both the United States and United Kingdom [4, 5]. Although additional validation studies are needed to establish how the self‐reported drinking temperature categories used here relate to absolute drinking temperatures, the available evidence suggests that increases in risk due to higher preferred drink temperatures may still be observed at temperature ranges below those cited in the IARC report (> 65°C). This is further supported by the high proportion of participants who added milk to their coffee and tea in the MWS, which is likely to be associated with lower consumed temperatures. While drink temperature and intake were assessed at a single time point, re‐analysis of 12‐year resurvey data in the MWS showed that preferences were generally stable over time, with over 60% of women reporting similar behaviours to those recorded at 8 years. Although the prospective design eliminates the potential for biased recall of exposures in those with a diagnosis of oesophageal cancer, it remains possible that preclinical symptoms affected behaviours and reporting of drink temperature at baseline, which could have biased the observed associations. However, analyses excluding the first 5 years of follow‐up yielded similar results to those observed overall.

## 5 Conclusions

This large study provides robust evidence that drink temperature as well as frequency of hot drink consumption are likely to be substantial risk factors for SCC. Our findings suggest that reducing drink temperature in populations where tea and coffee are frequently consumed could offer an important means of SCC prevention.

## Author Contributions

**Keren Papier:** conceptualisation, investigation, funding acquisition, methodology, formal analysis, writing – original draft, writing – review and editing, visualisation. **Kezia Gaitskell:** writing – review and editing, methodology. **Sarah Floud:** methodology, writing – review and editing. **Gillian K. Reeves:** conceptualisation, investigation, funding acquisition, writing – review and editing, methodology.

## Funding

The Million Women Study is funded by Cancer Research UK (grant number C16077/A29186) and UK Medical Research Council (grant number UKRI1464). K.P. is supported by the Nuffield Department of Population Health Intermediate Fellowship, University of Oxford. K.G. is supported by a UK Research and Innovation Future Leaders Fellowship (grant number UKRI2330). The funders had no role in study design, data collection and analysis, decision to publish or preparation of the manuscript. For the purpose of open access, the authors have applied a Creative Commons Attribution (CC BY) licence to any Author Accepted Manuscript version arising.

## Ethics Statement

The Million Women Study was approved by the East of England‐Cambridge South Research Ethics Committee (97/5/001). All participants provided written informed consent. The UK Biobank Study was approved by the National Information Governance Board for Health and Social Care and the National Health Service Northwest Multicentre Research Ethics Committee (06/MRE08/65).

## Conflicts of Interest

The authors declare no conflicts of interest.

## Supporting information


Supplementary Materials and Methods:
**Figure S1:** Participant flow chart for MWS and UKB.
**Figure S2:** Risk of oesophageal squamous cell carcinoma by preferred drink temperature and number of drinks consumed (6+ vs. < 6) by level of adjustment for potential confounders in MWS and UKB participants.
**Figure S3:** Risk of oesophageal squamous cell carcinoma by preferred tea and coffee temperature in MWS participants with 12‐year follow‐up information stratified by addition of milk to hot drinks.

## Data Availability

Data from the MWS are available to bona fide researchers in accordance with the MWS Data Access Policy (https://www.ceu.ox.ac.uk/research/the‐million‐women‐study/data‐access‐and‐sharing/data‐access‐policy). This research has been conducted using the UK Biobank Resource under Application Number 67506. UK Biobank is an open access resource. Bona fide researchers can apply to use the UK Biobank dataset by registering and applying at http://www.ukbiobank.ac.uk/register‐apply. Further information is available from the corresponding author upon request.
