# Supplementary material for: Hot Drinks and Oesophageal Cancer: Prospective Analyses in Around 1 Million UK Adults
Source: Int J Cancer. 2026 Sep 8;159(12):2877–85. doi: 10.1002/ijc.70654 (PMC13688219; doi:10.1002/ijc.70654)
Supplement: Supplementary file 1 [file IJC-159-2877-s001.pdf]

Hot drinks and oesophageal cancer: prospective analyses in around 1 million UK adults

Keren Papier, Kezia Gaitskell, Sarah Floud, Gillian K. Reeves

Table of Contents

Supplementary Materials and Methods 2

Supplementary Figures 4

Figure S1. Participant flow chart for MWS and UKB 4

Figure S2. Risk of oesophageal squamous cell carcinoma by preferred drink temperature and number of drinks consumed (6+ vs <6) by level of adjustment for potential confounders in MWS and UKB participants 5

Figure S3. Risk of oesophageal squamous cell carcinoma by preferred tea and coffee temperature in MWS participants with 12-year follow-up information stratified by addition of milk to hot drinks 6

Supplementary References 7

Supplementary Materials and Methods

Exclusions

In both cohorts, participants were excluded from analyses if they had been diagnosed with any cancer (except non-melanoma skin cancer C44) before the baseline survey, which was taken to be the 8-year survey in the Million Women Study (MWS) and the recruitment survey in the United Kingdom Biobank (UKB). Participants who had missing information on tea or coffee consumption, who selected that they did not consume any hot drinks for their preferred drink temperature, or who had missing information on drink temperature or tea or coffee intake, were also excluded. We excluded women who took part in both the MWS and the UKB study from the MWS dataset for all analyses to avoid duplication in the analysis dataset (Figure S1).

Adjustments

For MWS, analyses were stratified by year of birth, and region of residence (10 regions) and adjusted for alcohol (0,<=5,6-10, or 11+ drinks per week, unknown), smoking (never, past, current and <15, current and 15+ cigarettes per day, unknown), interaction term for alcohol x smoking, BMI (<20, 20-22.49, 22.5-24.9, 25-27.49, 27.5-29.9, 30-32.49, 32.5-34.49, 35+, unknown), education (tertiary, secondary, technical, none, unknown), deprivation (quintiles, unknown), height (<160, 160-164, or 165 or more cm, unknown), use of menopausal hormone therapy (ever, never, unknown), ethnicity (white, other, unknown), strenuous exercise (rarely or never, once or less per week, more than once per week, unknown), parity (null, 1-2, 3+, unknown). Analyses with respect to adenocarcinoma (AC) were additionally adjusted for self-reported information on: use of Losec in the last four weeks (commonly used to treat indigestion) (yes, unknown), use of Ranitidine (commonly used to treat ulcers) in the past four weeks (yes, unknown), having reflux or heartburn (never/rarely, < once a week, once a week, >once a week, unknown), and having difficulty with swallowing (never/rarely, <once a week, once a week, >once a week, unknown). For UKB, analyses were stratified by year of birth, region of residence (10 regions), and sex, and adjusted for smoking (as in MWS), BMI (as in MWS), alcohol (<1,1-9,10-19,20+ g/day, unknown), interaction term for alcohol x smoking, height (sex-specific quintiles, unknown), physical activity (low, medium, high, unknown), MHT use (never, past, current, N/A, unknown), parity (null, 1-2, 3+, unknown), ethnicity (white, other, unknown), education (tertiary, secondary, technical, unknown or missing), and deprivation (quintiles, unknown). Analyses with respect to AC were additionally adjusted for self-reported regular use of Ranitidine (no, yes, unknown). In both studies, analyses of squamous cell carcinoma (SCC) also included an interaction term between smoking status and alcohol intake to take into account the differing effect of smoking on SCC risk by alcohol intake[1]. All adjustment variables included an 'unknown' category for missing data. In MWS, all adjustment variables had <5% missing except for frequency of reported difficulty swallowing, and frequency of reported heartburn, for which the proportion with missing data ranged from 12%-27%. In UKB, all adjustment variables had <10% missing except for physical activity which had ~20% missing values.

Population attributable fractions

We estimated the proportion of cancers that would be avoided under a given scenario by calculating the population attributable fraction (PAF)[2] using the standard formula:

$$PAF = \frac{\sum_{i=1}^{n} p_i RR_i - \sum_{i=1}^{n} p_i' RR_i}{\sum_{i=1}^{n} p_i RR_i}$$

Where p^i (^{i}=1,…,n) denotes the proportion of the population exposed to the ith exposure category, $p^{i\prime}$ (^{i}=1,…,n) denotes the counterfactual proportion of the population exposed to the ith exposure category under a given scenario, and RR^i (^{i}=1,…,n) denotes the RR associated with each exposure level relative to the reference group (i.e. RR^1=1).

In this case, estimates of the proportions of the population exposed to each of the three preferred drink temperature categories were derived from self-reported information on preferred drink temperature in UK Biobank (men and women combined): 15.8% reported consuming warm drinks, 67.0% hot drinks, and 17.2% very hot drinks. The relative risks of SCC oesophageal cancer associated with each category of preferred drink temperature, relative to

warm, after adjustment for frequency of drink consumption and other potential confounders, were assumed to be 3.17 (2.49,4.03) (very hot) and 1.69 (1.36, 2.11) (hot) (see Figure 3 and Results p.3).

Based on these assumptions, the proportion of cancers that would be avoided if individuals who consumed their drinks at a very hot temperature instead consumed them at a hot temperature is estimated to be 14%.

In 2024, 9,437 UK adults were diagnosed with oesophageal cancer[3]. Assuming that approximately one third of these cases are SCC, this corresponds to around 3,146 new SCC cases annually[4]. Based on the estimated population attributable fractions given above, approximately 440 cases (14%) could be prevented if individuals who consume their drinks at a very hot temperature instead consumed them hot.

Supplementary Figures

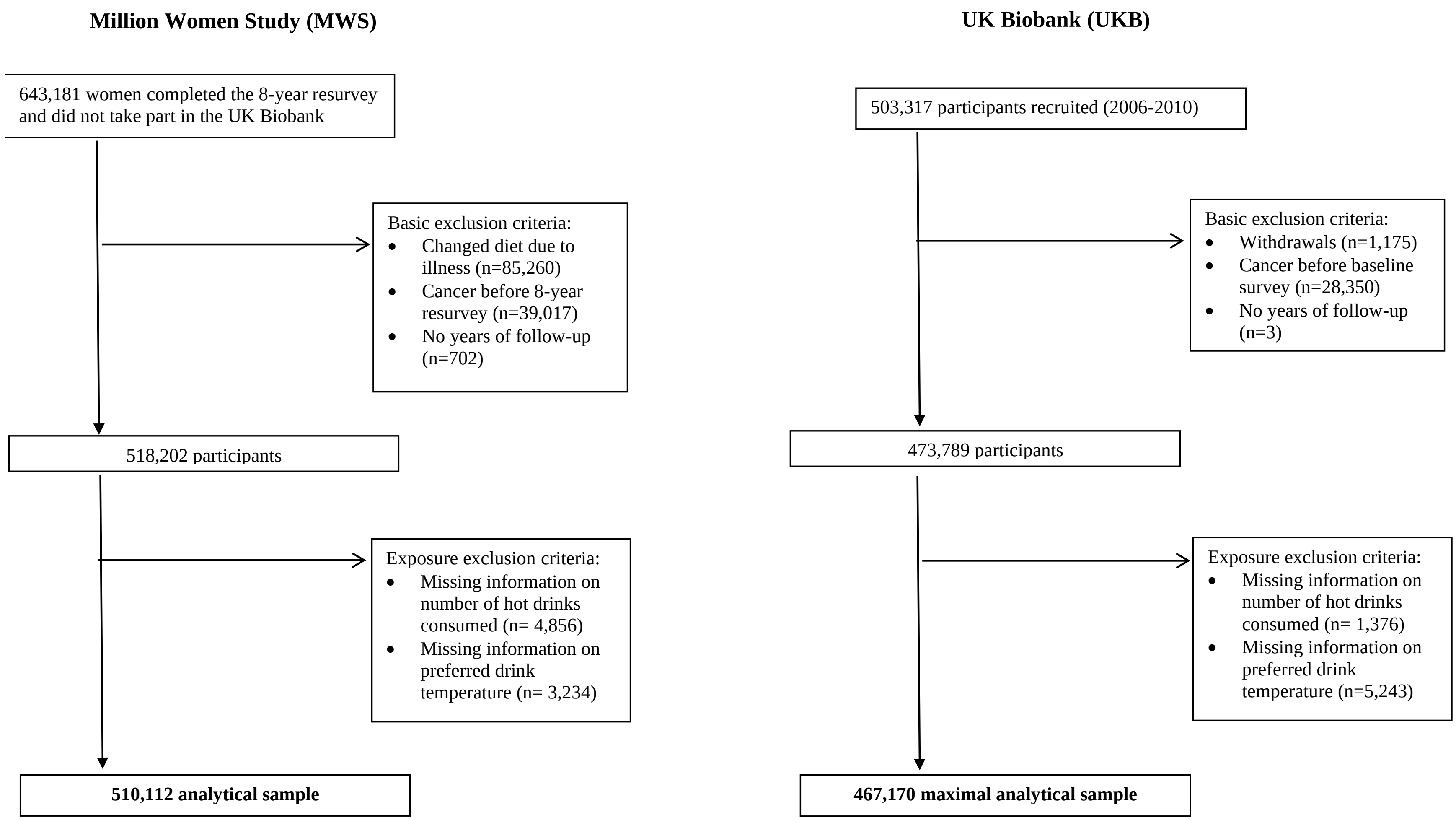

Figure S1. Participant flow chart for MWS and UKB

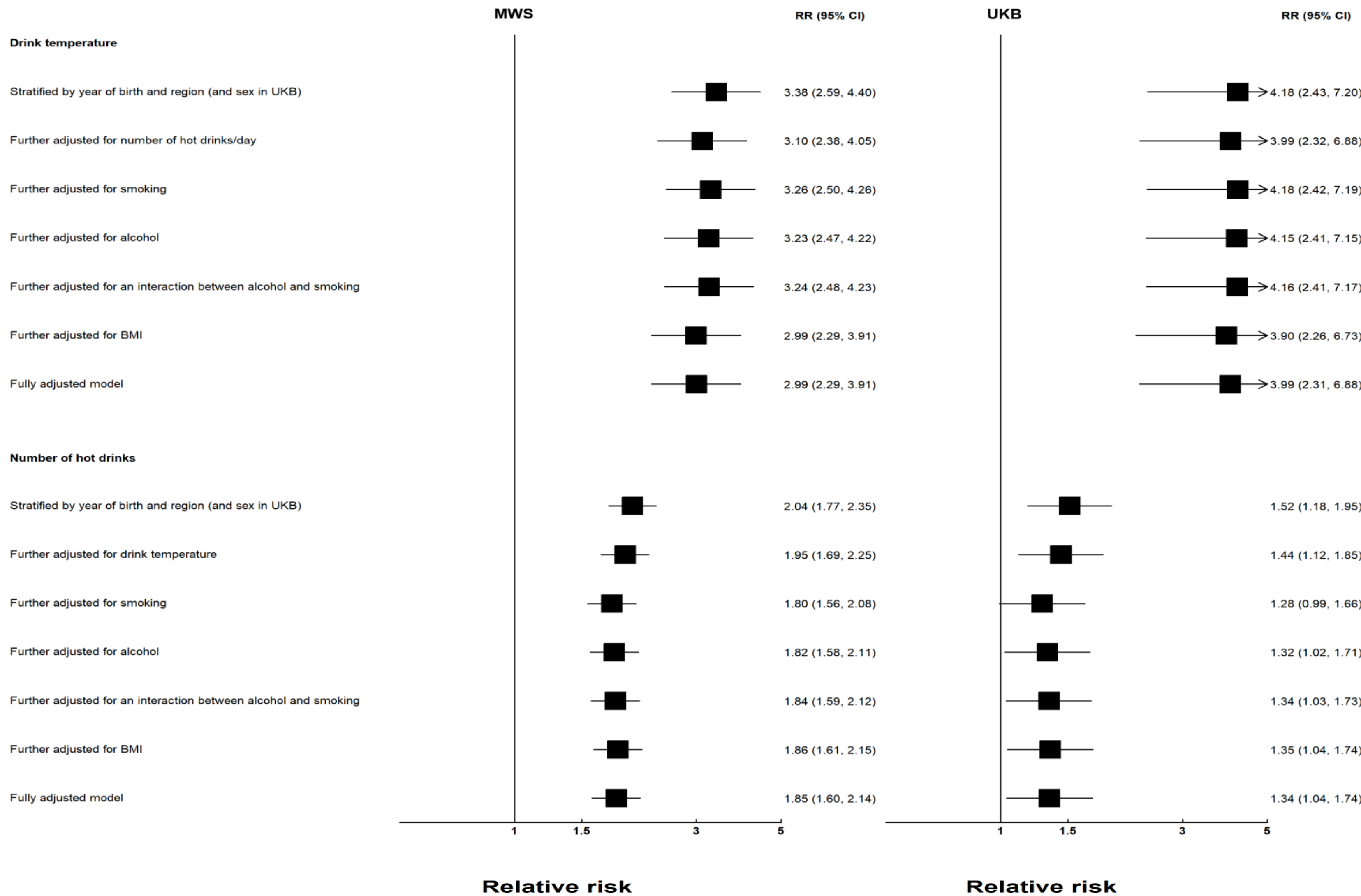

Figure S2. Risk of oesophageal squamous cell carcinoma by preferred drink temperature and number of drinks consumed (6+ vs <6) by level of adjustment for potential confounders in MWS and UKB participants

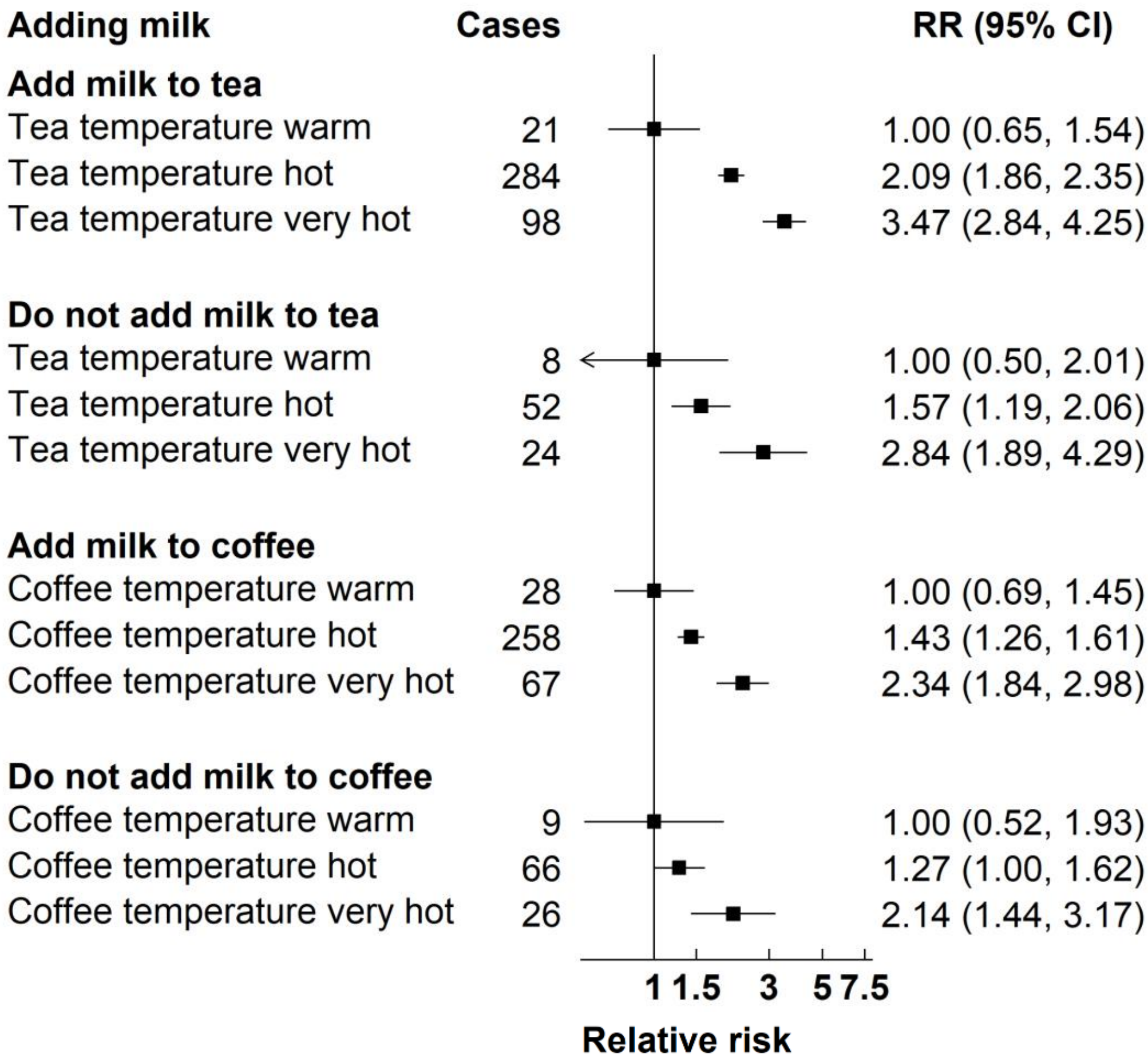

Figure S3. Risk of oesophageal squamous cell carcinoma by preferred tea and coffee temperature in MWS participants with 12-year follow-up information stratified by addition of milk to hot drinks

Tea and coffee temperature asked about separately at 12-year re-survey. Tea temperature data available for 458,316. Coffee temperature data available for 458,576 participants.

Supplementary References

1. Sweetland S, Floud S, Gaitskell K, Reeves GK. Adiposity and risk of oesophageal cancer subtypes in the Million Women Study. *Int J Epidemiol*. Dec 25 2023;52(6):1795-1804. doi:10.1093/ije/dyad094
2. Mansournia MA, Altman DG. Population attributable fraction. *Bmj*. 2018;360
3. World Cancer Research Fund. Oesophageal cancer. Accessed 13 April 2026, https://www.wcrf.org/preventing-cancer/cancer-types/oesophageal-cancer/
4. Cancer Research UK. What is oesophageal cancer? Accessed 13 April 2026, https://www.cancerresearchuk.org/about-cancer/oesophageal-cancer/about
